# Exploring feasibility of fNIRS to assess delayed inhibition effect of prefrontal cortex for acute stress by acupuncture on GV20: a pilot study

**DOI:** 10.3389/fnhum.2024.1433312

**Published:** 2024-11-15

**Authors:** Hideaki Tamai, Shoichi Komine, Senichiro Kikuchi, Hideaki Waki

**Affiliations:** ^1^Department of Acupuncture and Moxibustion, Faculty of Health Care, Teikyo Heisei University, Toshima-ku, Tokyo, Japan; ^2^Research Institute of Oriental Medicine, Teikyo Heisei University, Toshima-ku, Tokyo, Japan; ^3^Department of Anesthesiology and Critical Care Medicine, Jichi Medical University, Shimotsuke, Tochigi, Japan; ^4^Institute of Medicine, University of Tsukuba, Tsukuba, Ibaraki, Japan; ^5^Department of Rehabilitation Sciences, Graduate School of Health Sciences, Gunma University, Maebashi, Gunma, Japan

**Keywords:** stress, acupuncture, prefrontal cortex, cerebral blood flow, function near-infrared spectroscopy, ventrolateral prefrontal cortex

## Abstract

**Introduction:**

Acupuncture on the acupuncture point GV20 has been considered effective for managing psychoneurological disorders since ancient times, and GV20 is now regularly used to treat stress-related diseases (SRDs), including psychoneurological disorders. Although reports indicating that acupuncture stimulation of GV20 alone regulates cerebral blood flow (CBF) in brain regions associated with SRDs have been scattered, from the perspective of the need for earlier action, the effects on subjective stress as self-reported in the stress state of healthy individuals and CBF changes in related prefrontal cortex (PFC) subregions, which is based as underlying mechanism, remain unclear. Therefore, there is a need to explore effective methods for analyzing such effects.

**Methods:**

Multiple consecutive mental arithmetic tasks were performed to induce sufficient stress in healthy university students. Real acupuncture or sham acupuncture was performed on GV20, and subjective stress and CBF changes in PFC subregions were observed before and after acupuncture using functional near-infrared spectroscopy, which were analyzed based on the general linear model method and correlation analysis.

**Results:**

Subjective stress was suppressed and recovered significantly faster in the true acupuncture group than in the sham acupuncture group. Furthermore, significant negative correlations were found between subjective stress and CBF in several PFC subregions during and after the tasks, with more correlated regions on the left side than on the right side of the PFC. Among them, the CBF of the left ventrolateral PFC (vl-PFC) was suggested to be maintained in the true acupuncture group under repeated tasks load, which was inferred to be correlated with delayed decreases in subjective stress after acupuncture.

**Discussion:**

This pilot study showed that fNIRS is feasible for assessing delayed PFC inhibition in acute stress by acupuncture on GV20. Acupuncture treatment on GV20 resulted in early suppression of subjective stress and early recovery. The neuroscientific rationale for this finding may lie in its effect on maintaining CBF in the left vl-PFC related to delayed inhibition of subjective stress, and would make it reasonable to apply acupuncture to GV20 in healthy individuals. Larger studies are needed to corroborate these findings and obtain reliable conclusions.

## 1 Introduction

Stress refers to a state of threatened homeostasis provoked by a psychological, environmental, or physiological stressor (Liu et al., 2017). Evidence accumulated by many studies in the last two decades has indicated that severe or prolonged stress increases the risk of physical and psychiatric disorders, which are called stress-related diseases (SRDs) (Liu et al., 2017). Common SRDs include not only cardiovascular and metabolic diseases but also psychological and cognitive disorders (Chrousos, 2009; Guo et al., 2022). Therefore, stress is recognized as a major problem that leads to many diseases.

The brain plays a central role in stress reactivity, coping, and recovery processes (Mcewen and Gianaros, 2010). The brain systems that deal with stress include the prefrontal cortex (PFC), amygdala, and hippocampus (Mcewen and Gianaros, 2010). These systems regulate physiological and behavioral stress processes, which can be adaptive in the short term (acute stress process) and maladaptive in the long term (chronic stress process), and their maladaptation increases the risk of SRDs (Mcewen and Gianaros, 2010). Therefore, being able to respond adequately to stress as early as possible is desirable.

The PFC can manage the top–down regulation of stress processes and has higher cognitive functions (e.g., working memory and executive control) (Mcewen and Gianaros, 2010). Delayed and immediate responses are seen in response to acute stress in the PFC (Wang et al., 2005; Takizawa et al., 2014; Rahman et al., 2021). Specifically, activities in the PFC region immediately after acute stress correlate with self-reported subjective stress values (Fisch et al., 2020), and a correlation may exist between the response of several PFC regions and subjective stress values. This means that the stress process in the PFC continues even during the peripheral transmission of top–down instructions from the PFC. In contrast, chronic stress induces neurodegeneration and reduces activity in the PFC, which may shift from top–down to bottom–up information processing and enhance instinctive brain activity related to stress (Reser, 2016).

Acupuncture has been reported to be an effective treatment modality with moderate- or high-certainty evidence for not only musculoskeletal pain, intractable pain disorders, gynecological disorders, cranial nerve disorders, and allergic disorders, among others, but also mental illness (Lu et al., 2022; Chen et al., 2023). A study reported that the antidepressant, antianxiety, and other therapeutic effects of acupuncture are mediated through the central nervous system, which is involved in emotional and cognitive functions and physiological adjustments in the body (Fang et al., 2009). Considering the recent increase in the number of mental illnesses, providing acupuncture treatment at the stress state stage before the onset of illness would be desirable (Bower et al., 2023; Smrdu et al., 2023).

Various acupuncture points are used to treat SRDs. The BaiHui (GV20) acupoint has been usually used to treat stress-related neurological and psychiatric diseases, including depression (Deng et al., 2016; Duan et al., 2020; Zhou et al., 2022). Animal and human studies have reported that the antidepressant effect, recovery of cognitive function, and recovery of insomnia of acupuncture stimulation combining GV20 and other acupoints or electro-acupuncture stimulation on GV20 are associated with molecular biological, immunohistochemical, and brain physiological mechanisms of action (Deng et al., 2016; Duan et al., 2020; Li et al., 2021; Zhao et al., 2022; Yujuan et al., 2023; Zhang et al., 2023; Zheng et al., 2023). These findings suggest that acupuncture treatment on GV20 is effective in alleviating stress, which is the preliminary stage of SRDs. However, the effect and underlying neuroscientific mechanism of acupuncture using GV20 alone for treating stress has not been revealed.

Cerebral blood flow (CBF), which reflects brain activation and can be measured using functional brain imaging equipment, has been used as an objective indicator of psychological stress in humans (Wang et al., 2005; Takizawa et al., 2014). According to traditional Chinese medicine, GV20, which is on the “Du meridian,” governs blood vessels (Zhao et al., 2022). Therefore, it may be appropriate to evaluate the antistress effect of acupuncture on GV20 using indicators such as CBF. Therefore, investigating the effects of acupuncture using GV20 alone for treating stress and the neuroscientific mechanisms of action on it (including delayed effects on the PFC) would be meaningful.

The Uchida–Kraepelin test (UKT) is a useful method for simulating healthy individuals' pre-disease condition induced by mental arithmetic, a psychological stress task (Baba et al., 2021; Fujino et al., 2022; Oriyama, 2023). It is widely used worldwide. Several studies on neuroimaging evidence related to stress response induced by the UKT have been conducted (Higashi et al., 2004; Hamazaki-Fujita et al., 2011; Takizawa et al., 2014; Yamamoto et al., 2023). It is possible to consider the adaptation process of the acute and recovery phases of stress using the UKT. To the best of our knowledge, this is the first study using the UKT to investigate the effects of acupuncture on stress.

Functional near-infrared spectroscopy (fNIRS) is very useful for monitoring the CBF in the cerebral cortex during a mental workload experiment in daily environments (Quaresima and Ferrari, 2019). Therefore, fNIRS is an excellent tool for measuring brain function using stress-inducing tests, such as the UKT (subjects write down answers of serial mental arithmetic questions on a paper using a pencil on the sitting position).

This study aimed to explore the feasibility of using fNIRS, which assesses the effects of acupuncture on GV20 to inhibit and recover from acute stress induced by the UKT in healthy university students and to reveal the underlying mechanisms based on CBF changes in PFC subregions, which are related to immediate and delayed PFC inhibition in response to subjective stress. Therefore, three secondary hypotheses were developed in this study: first, verum acupuncture promotes either or both inhibition and recovery against subjective stress earlier than sham acupuncture; second, some PFC subregions are related to delayed inhibition of subjective stress in response to UKT tasks, under two types of acupuncture interventions; and third, verum acupuncture on GV20 facilitates CBF changes in any PFC subregion related to delayed inhibition of subjective stress.

## 2 Materials and methods

### 2.1 Participants

Fifteen right-handed healthy university students (10 males and five females) with a mean age of 21.6 ± 1.24 years participated in this study. They were recruited via fliers/posters at Teikyo Heisei University between July 7, 2021, and September 8, 2021. Two of the 15 subjects were excluded from this study because of their poor physical condition.

All participants were Japanese and were screened for the following inclusion criteria: (1) aged between 18 and 40 years and (2) right-handed. We used the H.N. Handedness Inventory to evaluate each subject's handedness. The exclusion criteria were as follows: (1) individuals aged below 18 years; (2) those with hemophilia or other diseases that present a bleeding tendency; (3) those with psychiatric or cranial nerve diseases and taking central nervous system stimulants or suppressants (e.g., sleeping pills, antidepressants, and antianxiety drugs); (4) those receiving acupuncture and moxibustion treatment; (5) smokers; (6) those with drinking habits; and (7) breastfeeding or pregnant individuals.

All participants refrained from strong-intensity physical activities, drinking alcohol, and unusual life activities the day before and the day of the experiment and from taking caffeinated beverages the day of the experiment. The participants were instructed to finish eating at least 3 h before the intervention.

This study was approved by the Ethics Committee of Teikyo Heisei University (approval number: 30-052-2). We obtained written informed consent from the subjects before all experiments.

### 2.2 Experimental design

**Figure 1:**
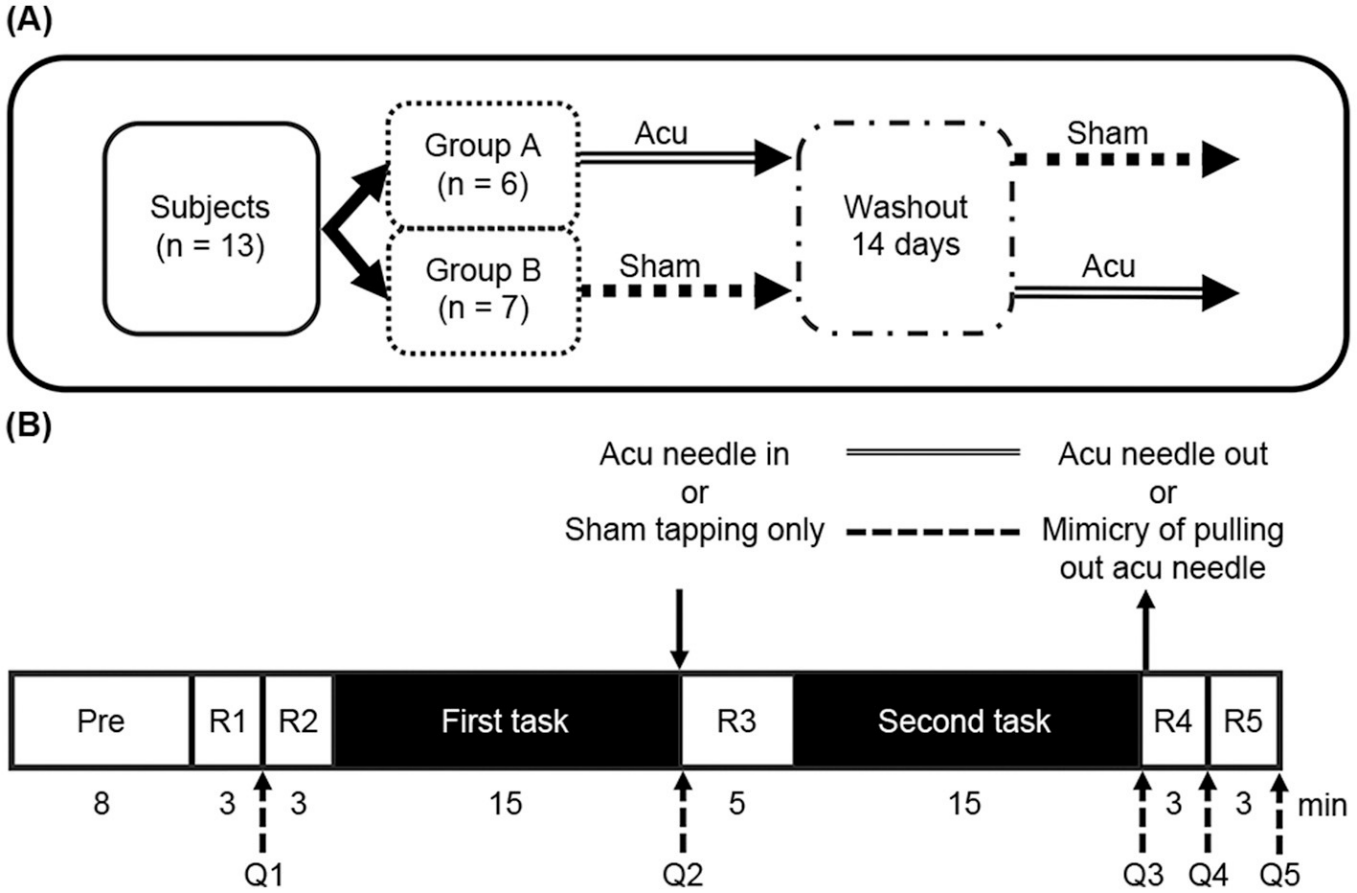
**(A)** Flowchart of the study design. **(B)** This study's protocol. Pre: preparation of this experiment including explain, setting the NIRS probe on the forehead and practice of the UKT; R: resting time; Q: questionnaire timing.

This study was designed as a randomized, controlled, and crossover trial with repeated measures for the intervention of verum or sham acupuncture treatment on GV20 (Figure 1A). First, the participants were randomly assigned to Group A or B. The experiments were performed two times in each group, and the washout period was 14 days. The two types of intervention were verum acupuncture and sham acupuncture, and the order of intervention was changed in each group.

### 2.3 Protocol

The protocol is shown in Figure 1B. First, in the preparation (Pre) stage, the participants sat in a chair and had the headband of the fNIRS device placed on their forehead (PFC area). They were then explained about the precautions for the experiment, as follows: do not keep eyes closed, keep them open normally, do not raise eyebrows, and do not tilt head or move body too much to prevent artifacts occurring in fNIRS data. Next, the procedures of the UKT were explained using a CD in voice, which included mental arithmetic practices that last for ~30 s (these practices are repeated three times). The preparation stage took ~8 min. After the preparation stage, 3 min of rest (R1) was provided in which the PC screen in front of the participants displayed a white fixation cross with a black background. The same was performed for R2, R3, R4, and R5. However, in R3, a 5-min rest was provided. After the preparation stage, the participants were instructed to indicate their subjective stress level using a Visual Analog Scale (VAS) (from 0 (no stress) to 100 (maximum stress)) (Fisch et al., 2020) as the first questionnaire (Q1). The same was performed in Q2, Q3, Q4, and Q5. After R2, the first task of the UKT was administered in 15 min, followed by the second task. After the first task, Q2 was performed, and verum or sham acupuncture was performed on the participants' GV20 location. After R3, the second task was administered. Q3 was then performed, and the verum acupuncture needle was pulled out or a mimicry of pulling out the acupuncture needle was performed. After R4, Q4 was performed. Following R5, Q5 was performed.

During the experiment, the participants sat on a comfortable chair quietly with their jaw placed on a chinrest in an air-conditioned room whose temperature was maintained at ~26.4°C ± 1.24°C and 26.0% ± 5.29% humidity.

### 2.4 Verum and sham acupuncture

Verum acupuncture as the real acupuncture treatment was performed on GV20, which is located at the highest place of the head with reference to the World Health Organization (WHO) acupuncture point positioning composition (World Health Organization (WHO) Regional Office for the Western Pacific Region, 2008). Verum acupuncture was performed according to the following procedure. A disposable acupuncture needle (Seirin, Shizuoka, Japan; length, 40 mm; diameter, 0.20 mm) was inserted at a depth of ~15 mm, and the needle was placed for ~20 min. Sham acupuncture was performed using the same procedure as the verum needles, except that the real needle was not present: the bottom of the plastic tube for needle insertion was contacted at the GV20 position, secured using the fingers of the left hand, and the other end of the tube was tapped several times with the second finger of the right hand.

### 2.5 Mental workload and psychological assessment

The UKT is a mental stress-inducing task used to assess the effect of the mental stress induced and to measure mental arithmetic work performance (Sugimoto et al., 2009; Yoto et al., 2018). We purchased standard test sheets (Nisseiken Inc., Tokyo, Japan). The UKT's preprinted paper comprises two tasks: one task with 15 lines, listing random single-digit numbers per line. The subjects were instructed to consecutively sum two adjacent digits up and write down the answers below each line for 1 min as soon and accurately as possible, and each task took 15 min. They are instructed to move to the next line according to the CD navigator's cue. As a psychological assessment, self-reported subjective stress was measured using a 100-mm VAS, as described above. Stress is a multifaceted concept, and its perception varies among individuals. In this study, we did not provide participants with a detailed definition of stress; instead, we used the term “stress” directly, as in previous research (Wang et al., 2005). Specifically, the questionnaire asked, “How stressed are you at the moment? Please mark the relevant part of the scale below [VAS scale from 0 (no stress) to 100 (maximum stress)].” Therefore, physical and mental stress was effectively assessed in this study. As a supplementary measurement, based on the number of answers and the number of incorrect answers obtained from each line of the UKT, the mean number of correct answers for each line was derived as the task performance value (Takizawa et al., 2014).

### 2.6 fNIRS measurement

**Figure 2:**
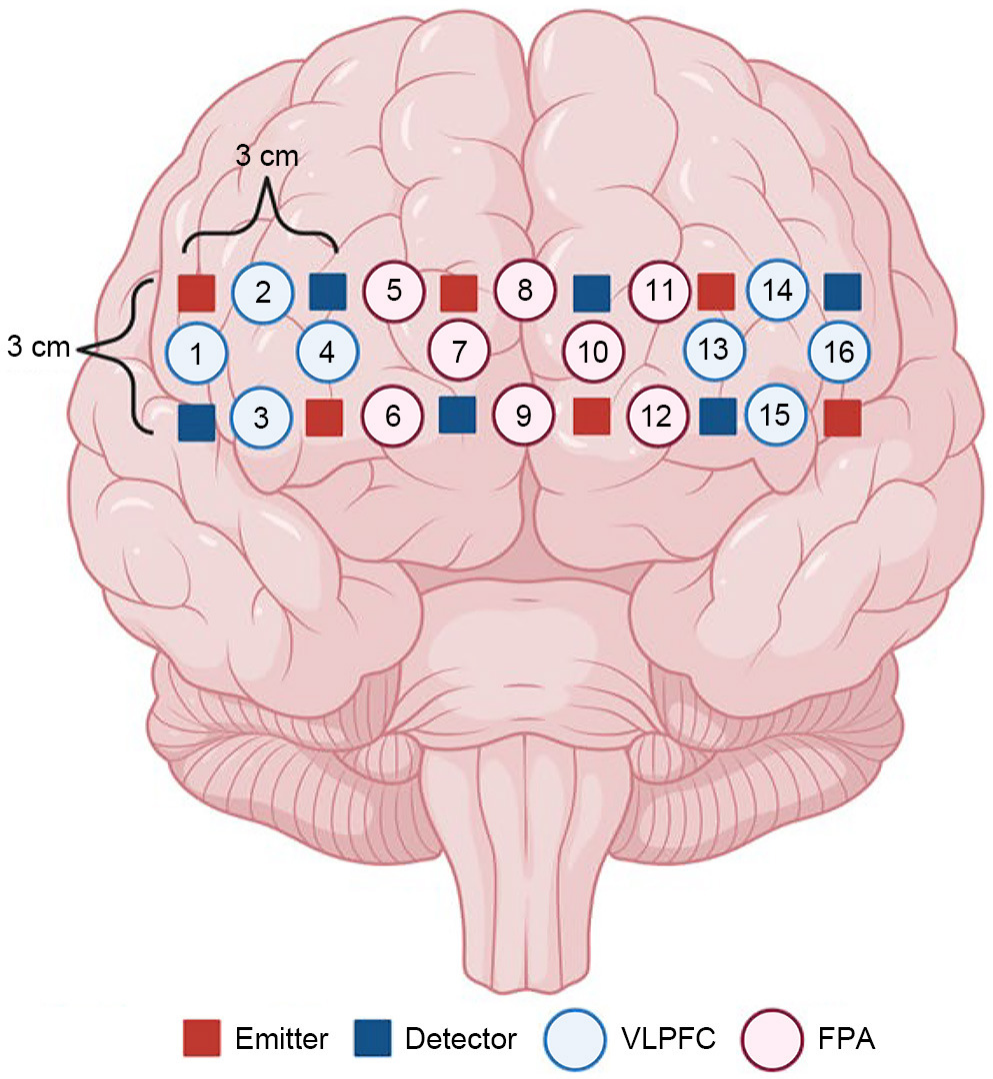
fNIRS channel placement according to the international 10–20 system. fNIRS channels were placed on two lateral PFC subregions named ventrolateral PFC (eight blue circles) and frontopolar area (eight red circles). There were 16 fNIRS channels. Created with BioRender.com.

Cerebral blood oxygenation in the PFC was measured using the 16-channel fNIRS system OEG-16 (Spectratech Inc., Tokyo, Japan). Oxyhemoglobin (Oxy-Hb) and deoxyhemoglobin (Deoxy-Hb) levels were measured using two wavelengths (770 and 840 nm) of infrared light based on the modified Beer–Lambert law (Watanabe et al., 1996). The sampling time was 0.76 Hz to acquire the fNIRS signal. This system has symmetric probe sets of six emitters and six detectors, which were placed alternately and comprised 16 measurement points (the distance between the emitter and detector was 3 cm). The near-infrared light probe sets were located on the subject's scalp over the PFC region, with the lowest line of the probe positioned on the Fp1, Fpz, and Fp2 lines of the international 10–20 system (Sasaguri et al., 2015; Al-Shargie et al., 2017). The approximate location of each channel is shown in Figure 2. The probe sets were located over the frontopolar and ventrolateral areas of the PFC, as previously described (Al-Shargie et al., 2017). This measurement was performed during all experimental periods, including the explanation and pre-practices of the UKT.

### 2.7 fNIRS signal processing

We chose Oxy-Hb as the main outcome measure because previous studies have revealed that Oxy-Hb correlates strongly with brain activation (Strangman et al., 2002; Negoro et al., 2010).

We used the hemodynamic modality separation system (Yamada et al., 2012) included in OEG-16 V3.0 (Spectratech Inc., Tokyo, Japan) to obtain functional components for the current tasks by separating systemic components, including physiological fluctuations, from Oxy-Hb signals (Takahashi and Grove, 2023).

Because the Oxy-Hb data include physiological noise, such as respiration and heartbeat, we applied a low-pass filter (0.1 Hz) to remove them using BRANL (BRSystems Inc., Kanagawa, Japan).

Next, the filtered data were analyzed using the general linear model (GLM) analysis function of BRANL to derive the Oxy-Hb concentration change value (*β* value) of each of the 16 channels.

### 2.8 Sample size

The sample size was calculated based on previous fNIRS pilot studies (Zheng et al., 2019; Fernandez Rojas et al., 2019; Yu et al., 2020). The average sample size in those studies was 12. Using a standardized effect size of 0.5 and power of 0.8, the sample size calculation indicated that 10 participants were required to detect the effect (Whitehead et al., 2016). Given the crossover design, each participant underwent both acupuncture and sham conditions. To account for potential dropouts, a redundancy of 20% was included, resulting in the recruitment of 15 participants. The final analysis included 13 participants who completed all sessions. In addition, we estimated the necessary sample size for future research based on this pilot study's fNIRS data using G^*^power (3.1.9.7) and a paired *t*-test model.

### 2.9 Statistical analysis

The acupuncture stimulation effect on subjective stress (VAS values) was evaluated using one-way repeated-measures analysis of variance (ANOVA) with the stimulation condition (verum acupuncture or sham acupuncture) as the independent variable. Significant main effects were followed by Bonferroni correction for *post hoc* comparisons within each group. Effect sizes were determined using partial eta squared ($\eta_{\text{p}}^{2}$) as suggested by Cohen (1973), with interpretation guidelines as follows: 0.01 (small effect), 0.09 (medium effect), and 0.25 (large effect).

Considering the study's experimental timeline encompassing both mental workload and acupuncture stimulation effects at different time points, similar analyses were performed to assess time-series changes for both effects. Group comparisons at each time point (Questionnaires 1 through 5) were conducted using independent samples *t*-tests.

Changes in task performance and *β* values across the 16 channels from the first to the second task of the UKT within each group were analyzed using paired samples *t*-tests. Group comparisons were made using independent sample *t*-tests. Effect sizes were assessed using Cohen's *d*, with interpretation guidelines as follows: 0.2 (small effect), 0.5 (medium effect), and 0.8 (large effect).

We applied the false discovery rate (FDR) method (Benjamini and Hochberg, 1995) to control for false positives following paired *t*-tests analyzing *β* values. Given the preliminary nature of our study and the limited literature on the stress-inhibitory effects of acupuncture in healthy young adults, we report our results before and after FDR correction.

Pearson's and Spearman's correlation coefficients were used to explore associations between VAS values and the mean number of correct answers for each task of the UKT, VAS and *β* values across the 16 channels, and the mean number of correct answers for each task and *β* values across the 16 channels.

Statistical significance was set at *p* < 0.05 and *q* < 0.05 (FDR), and a trend at *p* < 0.10. All analyses, except for FDR correction, were performed using the Statistical Package for the Social Sciences (SPSS, version 28.0.0.0). FDR correction was performed using Microsoft Excel 2019.

## 3 Results

### 3.1 Changes in subjective stress in the verum and sham acupuncture groups

**Figure 3:**
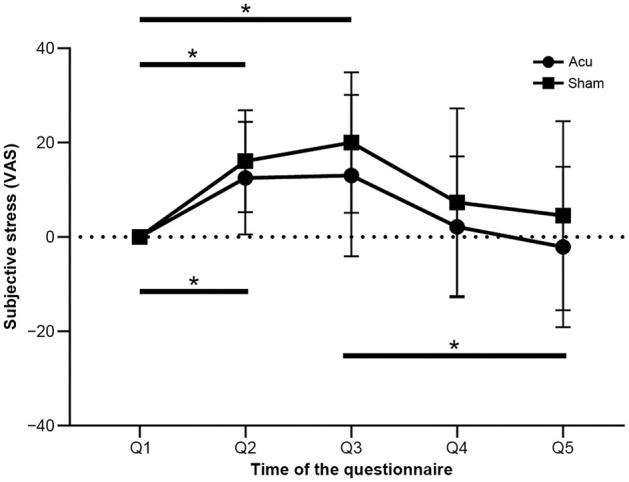
Changes in subjective stress of the verum and sham acupuncture groups during the experimental period. Error bars represent the standard deviation of the mean. *p < 0.05. Acu, verum acupuncture; Sham, sham acupuncture.

One-factor repeated-measures ANOVA and two-sample *t*-tests were performed to analyze the changes in subjective stress levels. Figure 3 displays the changes in subjective stress over the course of the experiments. The baseline stress level (Q1) was used as a reference point to assess the changes from Q2 to Q5.

The results of the one-factor repeated-measures ANOVA revealed significant differences in subjective stress levels within both the verum acupuncture group [*F*_(2.337, 28.042)_ = 5.780; *p* < 0.01; $\eta_{\text{p}}^{2}$ = 0.325] and the sham acupuncture group [*F*_(4, 48)_ = 7.327; *p* < 0.01; $\eta_{\text{p}}^{2}$ = 0.379]. *Post hoc* analysis using Bonferroni's correction indicated that the subjective stress level at Q2 significantly increased compared with that at Q1 in both the verum and sham acupuncture groups (*p* < 0.05). Furthermore, in the sham acupuncture group, the subjective stress level at Q3 significantly increased compared with that at Q1 (*p* < 0.05), whereas, in the verum acupuncture group, this increase was not significant. Furthermore, the subjective stress level at Q5 significantly decreased compared with that at Q3 in the verum acupuncture group (*p* < 0.05); however, no significant difference was observed in the sham acupuncture group. No significant differences or discernible trends in the changes were observed between the verum and sham acupuncture groups across individual time points from Q2 to Q5, as assessed by two sampled *t*-tests.

Overall, the results suggest that the first UKT task was effective in inducing subjective stress and that verum acupuncture can suppress subjective stress levels and facilitate quicker recovery than sham acupuncture. By measuring subjective stress levels at multiple time points (Q1–Q5), we were able to discern distinct patterns of stress modulation between the two acupuncture groups.

### 3.2 Correlations between subjective stress and CBF of PFC subregions

Correlations were analyzed using Spearman's correlation coefficient to investigate the relationship between subjective stress values from Q2 to Q5 and *β* values of the 16 channels of the PFC during the two UKT tasks. These analyses encompassed the combined results from both the verum and sham acupuncture groups (Table 1).

**Table 1:** Correlation coefficients between subjective stress and *β* values.

	**Q1**	**Q2**	**Q3**	**Q4**	**Q5**
**The first task** *β* **value**					
Ch2		−0.438^*^		−0.460^*^	−0.518^**^
Ch5				−0.397^*^	−0.439^*^
Ch11				−0.394^*^	−0.399^*^
Ch13			−0.351^†^	−0.409^*^	−0.435^*^
Ch14				−0.337^†^	−0.378^†^
**The second task** *β* **value**					
Ch2				−0.398^*^	−0.419^*^
Ch4			−0.346^†^		−0.352^†^
Ch5					−0.337^†^
Ch8				−0.432^*^	−0.454^*^
Ch11				−0.403^*^	−0.401^*^
Ch13			−0.433^*^	−0.362^†^	−0.360^†^
Ch14				−0.449^*^	−0.414^*^

Q1–Q5, five questionnaire timings. Values are expressed as Spearman's correlation coefficients.

^*^p < 0.05.

^**^p < 0.01.

^†^0.05 < p < 0.1.

#### 3.2.1 Correlations between *β* values of the first UKT task and subjective stress VAS values of Q2, Q3, Q4, and Q5

The *β* value of ch2 exhibited significant negative correlations with three VAS values: Q2 (rs = −0.438, *p* < 0.05), Q4 (rs = −0.460, *p* < 0.01), and Q5 (rs = −0.518, *p* < 0.01). Similarly, the *β* value of ch5 showed significant negative correlations with two VAS values: Q4 (rs = −0.397, *p* < 0.05) and Q5 (rs = −0.439, *p* < 0.01). Furthermore, the *β* value of ch11 demonstrated significant negative correlations with two VAS values: Q4 (rs = −0.394, *p* < 0.05) and Q5 (rs = −0.399, *p* < 0.05). The *β* value of ch13 exhibited significant negative correlations with two VAS values: Q4 (rs = −0.409, *p* < 0.05) and Q5 (rs = −0.435, *p* < 0.05) and a trend for negative correlation with a VAS value: Q3 (rs = −0.351, 0.05 < *p* < 0.1). Finally, the *β* value of ch14 exhibited a trend for negative correlations with two VAS values: Q4 (rs = −0.337, 0.05 < *p* < 0.1) and Q5 (rs = −0.378, 0.05 < *p* < 0.1).

#### 3.2.2 Correlations between *β* values of the second UKT task and subjective stress VAS values of Q3, Q4, and Q5

The *β* value of ch2 was significantly negatively correlated with two VAS values of subjective stress: Q4 (rs = −0.398, *p* < 0.05) and Q5 (rs = −0.419, *p* < 0.05). Similarly, the *β* value of ch4 showed a trend for negative correlation with two VAS values: Q3 (rs = −0.346, 0.05 < *p* < 0.1) and (rs = −0.352, 0.05 < *p* < 0.1). The *β* value of ch5 tended to be negatively correlated with the VAS value of Q5 (rs = −0.337, 0.05 < *p* < 0.1). Furthermore, the *β* value of ch8 demonstrated significant negative correlations with two VAS values: Q4 (rs = −0.432, *p* < 0.05) and Q5 (rs = −0.454, *p* < 0.05). Likewise, the *β* value of ch11 exhibited significant negative correlations with two VAS values: Q4 (rs = −0.403, *p* < 0.05) and Q5 (rs = −0.401, *p* < 0.05). Moreover, the *β* value of ch13 showed significant negative correlations with the VAS value of Q3 (rs = −0.433, *p* < 0.05), a trend for negative correlation with two VAS values of Q4 (rs = −0.362, 0.05 < *p* < 0.1), and Q5 (rs = −0.360, 0.05 < *p* < 0.1). Finally, the *β* value of ch14 demonstrated significant negative correlations with two VAS values: Q4 (rs = −0.449, *p* < 0.05) and Q5 (rs = −0.414, *p* < 0.05).

These results suggest that the number of activated prefrontal subregions related to stress inhibition increases over time when the present subjects are subjected to multiple continuous mental stress loads with intervals of a few minutes.

### 3.3 Difference in *β* values between the first and second tasks within the verum or sham group and that in each task between the verum and sham groups

**Figure 4:**
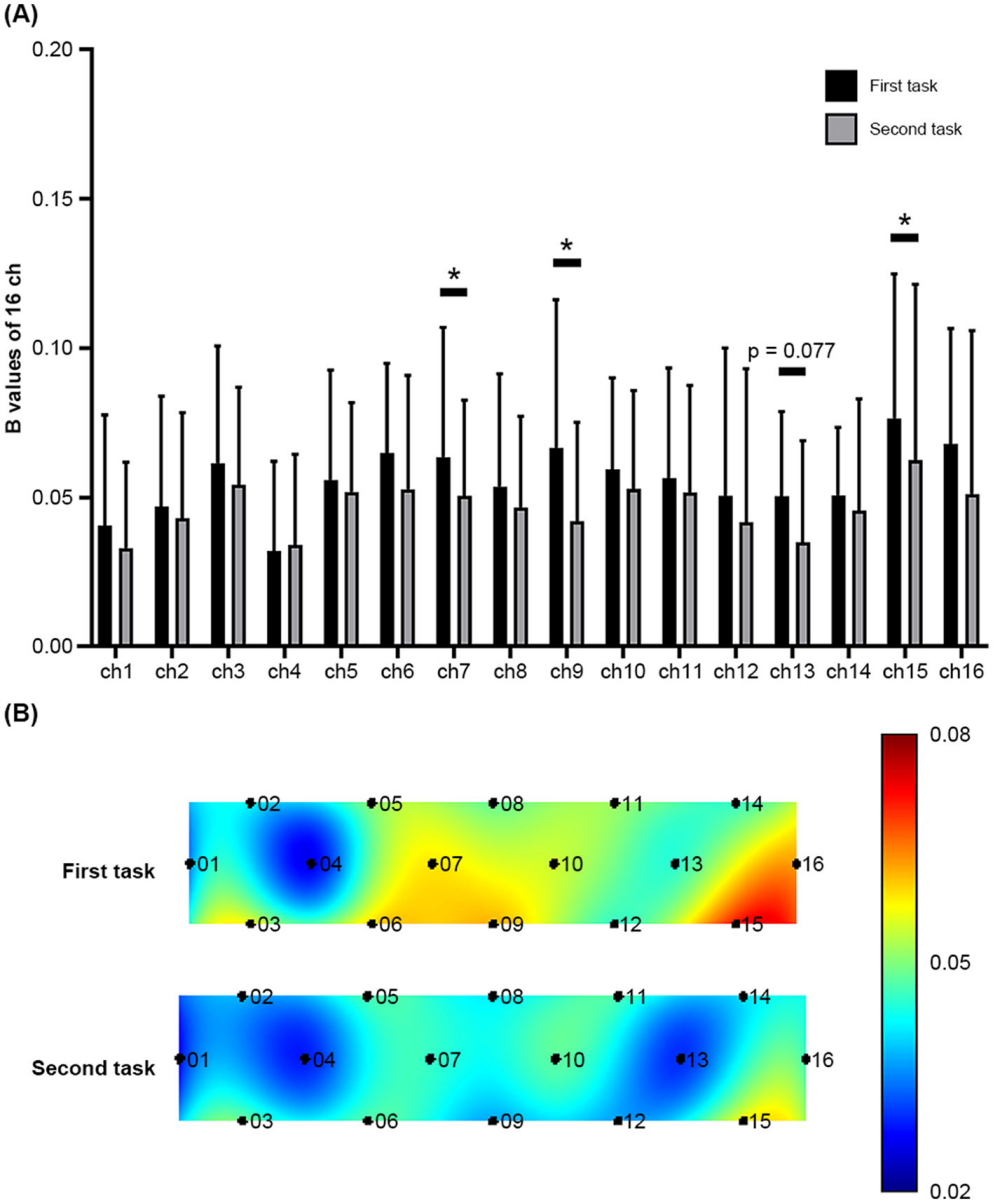
**(A)** The *β* values of Oxy-Hb of the first and second tasks across the 16 fNIRS measurement channels during sham acupuncture. Error bars represent the standard deviation of the mean. *p < 0.05. **(B)** Visualization of the *β* values in the PFC during the two tasks. Greater prefrontal activation as *β* values is represented by red color, whereas lower prefrontal activation is represented by blue color.

**Figure 5:**
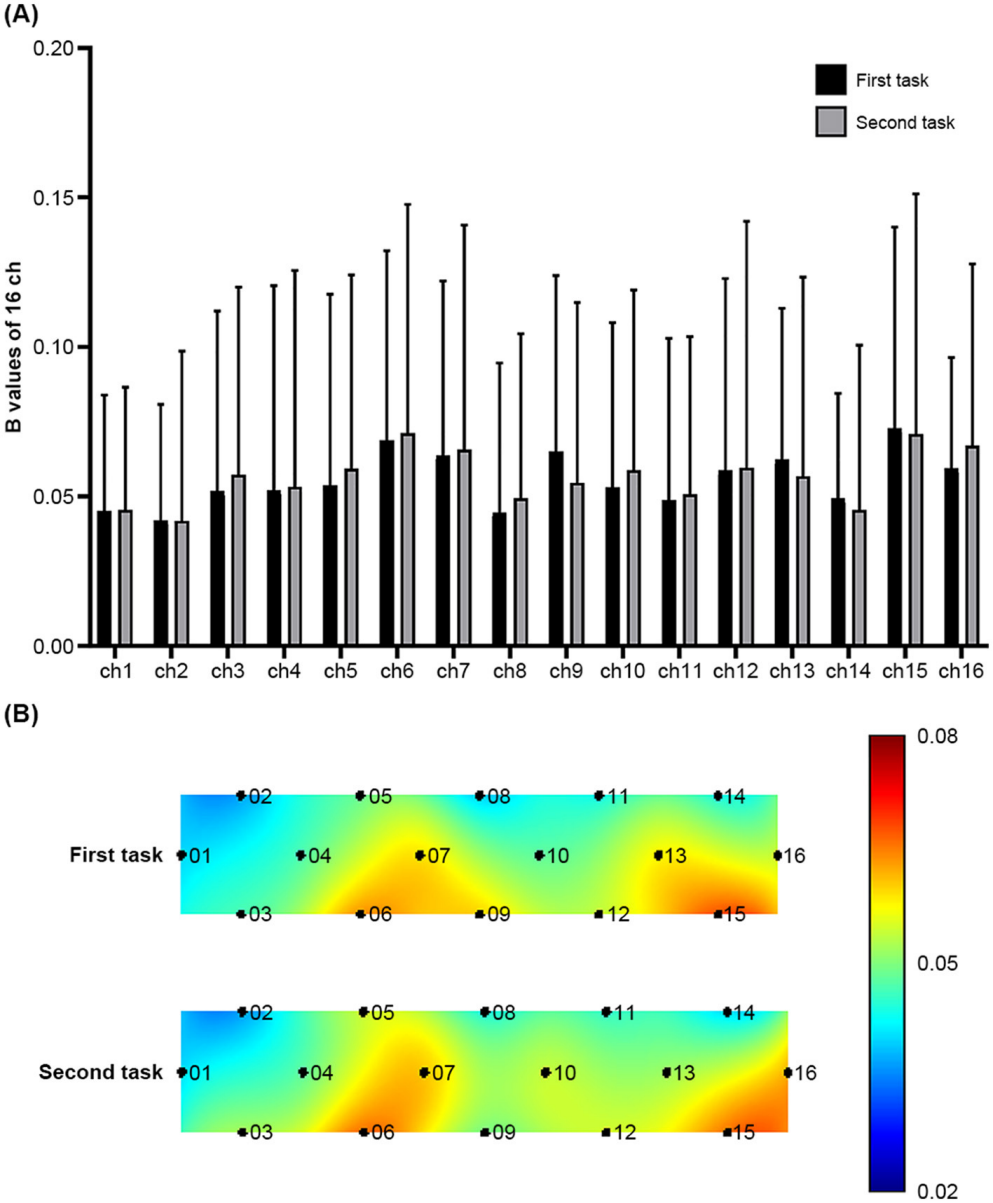
**(A)** The *β* values of Oxy-Hb of the first and second tasks across the 16 fNIRS measurement channels during verum acupuncture. Error bars represent the standard deviation of the mean. **(B)** Visualization of the *β* values in the PFC during the two tasks. Greater prefrontal activation as *β* values is represented by red color, whereas lower prefrontal activation is represented by blue color.

Paired *t*-tests and two-sample *t*-tests were performed to examine the difference in *β* values of the 16 channels between the first and second tasks within the verum or sham acupuncture group and the difference in those values for each task between the verum and sham groups. Figures 4, 5 illustrate the findings.

Within the sham acupuncture group, significant differences and a differential tendency were observed in ch7, ch9, ch13, and ch15 between the first and second tasks [(ch7: *t*_(12)_ = 2.460, *p* = 0.03, Cohen's *d* = 0.275), (ch9: *t*_(12)_ = 2.972, *p* = 0.012, Cohen's *d* = 0.506)] [(ch13: *t*_(12)_ = 1.937, *p* = 0.077, Cohen's *d* = 0.482), (ch15: *t*_(12)_ = 2.312, *p* = 0.039, Cohen's *d* = 0.230)], whereas no significant differences were observed within the verum group. Furthermore, no significant differences or tendencies were observed between the verum and sham groups for either the first or second task.

These results indicate that verum acupuncture may maintain the CBF of ch13, which is related to delayed inhibition of mental stress. However, after FDR adjustment, the sham acupuncture group did not show any significant difference or tendency ([ch7: FDR *q* = 0.24], [ch9: FDR *q* = 0.192]; [ch13: FDR *q* = 0.308]; and [ch15: FDR *q* = 0.208]). While these data confirm that our preliminary study was underpowered to detect significant differences, it provides important data for calculating the sample size needed to detect ch13 activation of vl-PFC in a future large-scale 16-channel fNIRS study.

### 3.4 Correlations between subjective stress and the mean number of correct answers

Correlations were assessed using Pearson's correlation coefficient. Table 2 presents the correlations between subjective stress values from Q1 to Q5 and the mean number of correct answers for both UKT tasks, including combined results from the verum and sham acupuncture groups.

**Table 2:** Correlation coefficients between subjective stress and the mean number of correct answers.

	**Q1**	**Q2**	**Q3**	**Q4**	**Q5**
The first task mean number of correct answers	−0.697^**^	−0.683^**^	−0.526^**^	−0.474^*^	−0.509^**^
The second task mean number of correct answers	−0.730^**^	−0.717^**^	−0.535^**^	−0.534^**^	−0.574^**^

Q1–Q5, five questionnaire timings. Values are expressed as Pearson's correlation coefficients.

^*^p < 0.05.

^**^p < 0.01.

A significantly negative correlation was found between all five subjective stress values and the mean number of correct answers for the first task (*p* < 0.05 and *p* < 0.01) in both groups. Similar correlations were observed between these stress values and the mean number of correct answers for the second task (*p* < 0.05 and *p* < 0.01).

This finding suggests that increased subjective stress decreases the brain's ability to respond correctly to mental arithmetic tasks of the UKT.

### 3.5 Mean number of correct answers in the UKT in the verum and sham acupuncture groups

In this study, we evaluated the mean number of correct answers in the UKT in both the verum and sham acupuncture groups. Paired *t*-tests and two-sample *t*-tests were performed to compare the differences in the mean number of correct answers between the first and second UKT tasks within each group.

**Figure 6:**
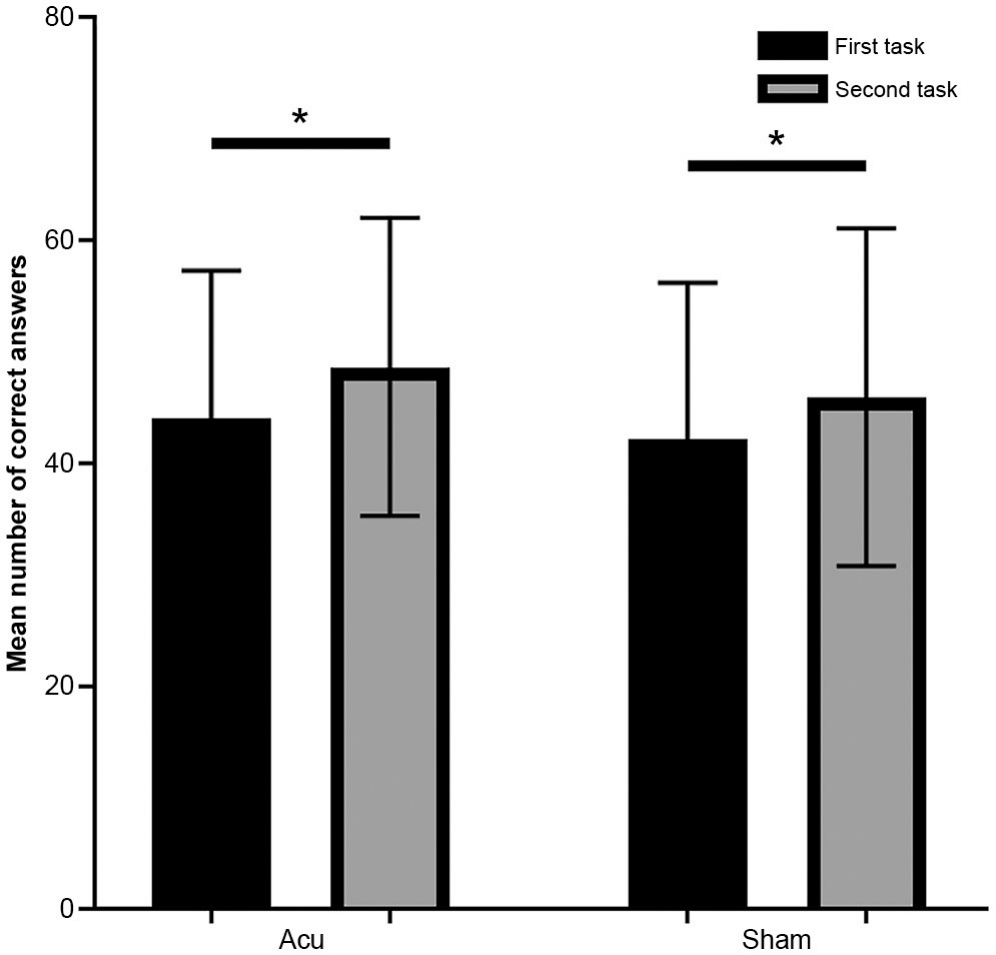
The number of mean correct answers in the first and second tasks in the verum and sham acupuncture groups. Error bars represent the standard deviation of the mean. *p < 0.05. Acu, verum acupuncture; Sham, sham acupuncture.

Within both the verum and sham acupuncture groups, the mean number of correct answers significantly increased more in the second task than in the first task of the UKT [verum acupuncture: *t*_(12)_ = −7.938, *p* < 0.01, Cohen's *d* = −0.341; sham acupuncture: *t*_(12)_ = −3.559, *p* < 0.01, Cohen's *d* = −0.249; Figure 6]. However, no significant difference or differential tendency was found between the two groups in terms of performance on the UKT tasks across the two time points.

This finding suggests that compared with sham acupuncture, verum acupuncture did not affect cognitive function related to correct answers to the mental arithmetic tasks of the UKT.

### 3.6 Correlations between the mean number of correct answers and *β* values of the PFC

Correlations were analyzed using Spearman's correlation coefficient. Table 3 presents trend of correlations between the mean number of correct answers and *β* values of the PFC (0.05 < *p* < 0.1).

**Table 3:** Correlations between the mean number of correct answers of the UKT and *β* values.

	**Mean number of correct answers in the first UKT task**	**Mean number of correct answers in the second UKT task**
**The second UKT** *β* **value**		
Ch3		0.386^†^

Values are expressed as Spearman's correlation coefficients.

^†^0.05 < p < 0.1.

#### 3.6.1 Correlations between the *β* values of the first task and the mean number of correct answers of the first and second tasks

There was no significant correlation or correlation trend between *β* values in the first task and the mean number of correct answers of both tasks (data not shown).

#### 3.6.2 Correlations between the *β* values of the second task and the mean number of correct answers of the second task

The *β* values of ch3 shows trend correlated with the mean number of correct answers of the second task (rs = 0.386, 0.05 < *p* < 0.1).

This result indicates that repeated mental arithmetic tasks make the right vl-PFC focused as the brain region responsible for mental arithmetic function in both verum and sham acupuncture groups.

### 3.7 Necessary sample size for future research

This pilot study allowed us to estimate the necessary sample size for future research. To reliably detect left vl-PFC activation in a 16-channel fNIRS study, we calculated that 63 participants would be required based on an effect size of 0.5, a power of 0.8, and an adjusted *p*-value of 0.003125 obtained from the FDR correction applied to this fNIRS data.

## 4 Discussion

The main aim of this study was to explore the feasibility of using fNIRS to assess the effects of verum acupuncture on GV20 on subjective stress and CBF in the PFC subregions of healthy university students. Three key findings support this aim, mostly aligning with our hypotheses.

First, verum acupuncture was found to exert a faster inhibitory effect on subjective stress levels and promote faster recovery than sham acupuncture. Second, under the two types of acupuncture interventions, multiple PFC subregions were found to be related to delayed inhibition of subjective stress in response to successive mental workload; furthermore, the number of these regions increased over time. Third, although this study was underpowered to detect significant differences, the potential effect of verum acupuncture on GV20 in facilitating CBF in the left ventrolateral PFC may be revealed from the delayed inhibition of subjective stress in a future large study.

The findings of this study indicate that acupuncture stimulation on GV20 can promote the inhibition of subjective stress induced by mental workload. This agrees with the main idea of existing studies (Rosted et al., 2010) that emphasize the ability of acupuncture including GV20 to reduce mental stress. Furthermore, no reports have examined the underlying neuroscientific mechanisms in conjunction with the subject's subjective stress changes caused by acupuncture stimulation to GV20 alone (Rosted et al., 2010; Deng et al., 2016; Sakatani et al., 2016; Duan et al., 2020; Wei et al., 2021). Therefore, this study is the first to indicate the feasibility of using fNIRS to assess the delayed inhibitory effect of acupuncture on GV20 alone on subjective stress from a neuroscientific perspective.

The results of this study suggest that the mechanism of subjective stress inhibition and recovery is a combination of immediate and delayed stress inhibition effects through the activation of multiple PFC subregions. After a stressful load, the brain establishes bidirectional contacts with the autonomic, cardiovascular, and immune systems to promote short-term adaptation after some time (Mcewen and Gianaros, 2010). Moreover, it has been reported that there are time differences in the responses in different regions for stress adaptation in the brain (Becker and Rohleder, 2019). In this study, the number of PFC channels associated with delayed stress inhibition increased during the second task compared with that during the first task, suggesting that the number of PFC subregions associated with delayed stress inhibition increases during the second task compared with that during the first task in response to increased stress load. An animal study reported delayed and sustained effects of PFC neurons on acute stress (Yuen et al., 2011). However, to the best of our knowledge, this is the first report to expand the phenomenon of the area involved in the delayed inhibition of stress in human PFC subregions after multiple acute stress stimuli in the scale of a pilot study.

The number of left PFC subregions associated with delayed stress inhibition in the second task increased more than that of the right PFC subregions. Among the left PFC channels correlated with subjective stress VAS values, the *β* value of ch13 was lower during the second task than during the first task in the sham acupuncture group according to the stage of the paired *t*-test. It has been reported that in stress induced by mental arithmetic tasks, the right vl-PFC activity activated during the stress load continues to be activated after the stress load ends; however, simultaneously, activation of the left vl-PFC is reduced (Wang et al., 2005). The results of the sham acupuncture group with significant difference that could be observed in the future large study may be consistent with the results of this study in that CBF in the left vl-PFC decreases under stress conditions (Wang et al., 2005). However, in the verum acupuncture group, it was maintained at the same level from the first task to the second task, suggesting that ch13 may play an important role in delayed stress inhibition after the first and second tasks. This expected result is similar to those of previous studies from the perspective that the left PFC is important for stress suppression (Tanida et al., 2008; Ishikawa et al., 2014).

Regarding changes in resting-state brain functional connectivity induced by acute stress, the amygdala plays a central role in adaptation during the onset and recovery phases of acute stress and correlates with several PFC subregions (Quaedflieg et al., 2015). Furthermore, the left vl-PFC showed reduced activity correlated with the amygdala in response to acute stress (Quaedflieg et al., 2015). This study showed that the effects of acute stress-induced activity changes in the left vl-PFC correlated with subjective stress continue not only in the immediate period but also in the recovery period. Therefore, ch13 in the left vl-PFC may be considered a key region of the amygdala-centered network that works for stress inhibition and may be important in the delayed stress inhibition effect of acupuncture on GV20 in this study. The expectation that a sustained effect may be observed in brain activity after acupuncture on GV20 is similar to a previous study (Wei et al., 2021). Moreover, the possible number of regions with decreased CBF after stress loading in the sham acupuncture group suggests a possible physiological brain response to verum acupuncture stimulation.

We made auxiliary observations on task performance related to cognitive function and subjective stress. In this study, a significant negative correlation was found between the subjective stress VAS values from Q1 to Q5 and the mean number of correct answers during the first and second tasks. It has been previously shown that acute stress reduces cognitive abilities, including working memory, and induces negative emotions, similar to our results (Gärtner et al., 2015; Picciotto and Fabio, 2024). Moreover, the mean number of correct answers was higher on the second task than on the first task. The UKT tasks were not difficult for the subjects in this study, and the mean percentage of correct answers was ~99% for both the first and second tasks (data not presented). The results of this experiment are similar to those of previous studies (Nakano and Kageyama, 2019, 2020), which found a delayed increase in the number of answers on the second time and conceived that healthy young Japanese individuals of the same age feel that the UKT tasks are not difficult, and the error rate was very low (average error rate of < 1%), which is considered to be similar to that of the previous study (Oriyama and Yamashita, 2021). Studies have shown that the left and right vl-PFC are important PFC subregions related to task performance on the UKT (Rivera et al., 2005; Takizawa et al., 2014), and the delayed increase in answers may be due to practice, which improves cognitive test scores due to repeated exposure to the same tests (McCaffrey and Westervelt, 1995; Duff et al., 2015), and the underlying mechanism may be the promotion of functional neuroplasticity in these regions (Li et al., 2020).

In this study, the repeated mental arithmetic task indicated that the region responsible for this function may be at ch3 (right vl-PFC). Despite a trend toward a correlation between the number of mean correct answers of the second UKT and the *β* value of ch3 during the second UKT, there was no significant difference in the *β* value of ch3 between tasks in both treatment groups. This indicates that the efficiency of the mental arithmetic function may be increased by functional neuroplasticity in the right vl-PFC. Both groups showed a similar increase in number of mean correct answers. The placebo effect on cognitive function, which may occur with sham acupuncture, is not well-studied. Recent reports indicate that when testing working memory, as in the UKT used in this study, the placebo effect is less likely to occur in healthy young adults (Blokland, 2023). Therefore, it is likely that in this study neither acupuncture nor sham acupuncture had a significant effect on cognitive function.

Studies have suggested that acupuncture including GV20 can improve cognitive function in healthy individuals and those with various cognitive impairments, including primary insomnia and CSVDCI, which is the most common cause of vascular cognitive impairment (Ren et al., 2021; Shuangjuan and Ximei, 2023; Yang et al., 2023). However, the acupuncture stimulation in these studies was combined with other acupuncture points, stimulated with electric current, retained in the body for a longer time, and performed longer days, suggesting that the amount of stimulation was greater than our acupuncture stimulation.

Although the possibility of acupuncture stimulation of GV20 alone improving cognitive function can be inferred, in the present study, there was no subregion, including ch3, which may be related to task performance as subregion whose CBF decreased in the second UKT compared with that in the first UKT in the verum acupuncture group. Therefore, our verum acupuncture stimulation appears inadequate for affecting cognitive function based on changes in CBF presented as neuroplasticity.

Thus, our findings support the feasibility of using fNIRS, which assesses the effects of acupuncture on GV20 for the inhibition of and recovery from acute stress in healthy university students and indicate that acupuncture on GV20 alone could be effective in inhibiting and recovering from mental stress in terms of CBF changes in PFC subregions. However, large-scale studies are needed to reveal the underlying mechanisms and confirm our findings.

## 5 Limitation

This preliminary study has five potential limitations. First, the number of subjects was relatively small because of a pilot study scale. To reliably detect left vl-PFC activation, a larger number of subjects (63 participants) are required in future studies. Second, we did not analyze prefrontal cortex activity during resting state before and after the UKT because we could not find an appropriate method for analyzing resting-state activity of the PFC, which could become equivalent to the GLM method, which analyzes activation state during the UKT. Third, we performed acupuncture stimulation once only in this study; however, acupuncture is generally performed many times, for example, several days consecutively or once a week, and is continued under clinical practice in many countries. Furthermore, such continuous acupuncture stimulation has been reported to have cumulative effects on the neuronal system (Li et al., 2014; Chen et al., 2022). Therefore, we may need to investigate the long-term effects of acupuncture on GV20 on neurophysiological changes in the future. Fourth, sham acupuncture, which involved placing a plastic tube against GV20 and tapping it several times, may have had a minor effect on its own. However, verum acupuncture would be able to produce verum acupuncture effects even in situations where that method is employed. Fifth, all participants were college students studying acupuncture, which means that they may be able to distinguish between real and sham stimuli than the general population. This may have influenced the subjective stress results and may represent selection bias.

## 6 Conclusion

This pilot study shows that fNIRS is a feasible setup to assess delayed PFC inhibition of acute stress using acupuncture on GV20. Our study indicates that acupuncture on GV20 may help inhibit and recover from subjective stress in healthy university students and may be brought about possibly by maintaining CBF of the left vl-PFC without decrease under repeated mental workload. Future large-scale research to reveal the underlying mechanisms of action of acupuncture on GV20 for stress inhibition and recovery, including reliable detection left vl-PFC activation, are needed. Our findings may help clarify the efficacy of the acupuncture point GV20 and provide one research method based on neuroscience to prove that acupuncture using a single acupuncture point can be effective.

## Data Availability

The datasets presented in this article are not readily available because the datasets used and/or analyzed during the current study are available from the corresponding author on reasonable request. Requests to access the datasets should be directed at: hideaki.tamai@thu.ac.jp.
